# Effects of calf muscle conditioning upon ankle proprioception

**DOI:** 10.1371/journal.pone.0236731

**Published:** 2020-08-31

**Authors:** Raymond F. Reynolds, Craig P. Smith, Rufei Yang, Robert Griffin, Amanda Dunn, Craig McAllister

**Affiliations:** The School of Sport, Exercise & Rehabilitation Sciences, The University of Birmingham, Birmingham, United Kingdom; The Wingate College of Physical Education and Sports Sciences at the Wingate Institute, ISRAEL

## Abstract

Ankle proprioception is crucial for balance and relies upon accurate input from calf muscle spindles. Spindle input, in turn, depends upon the physiological and mechanical properties of surrounding muscle tissue. Altering these properties could affect ankle proprioception, with potential consequences for balance. Here we determine the effects of prior muscle cooling, stretch and contraction upon performance of a contralateral ankle joint matching task. Participants stood passively leaning against a board oriented 22° rearward from vertical. Their right ankle was rotated to a randomised position between ± 6° plantar/dorsiflexion. The task was to align the left ankle to the same position, without vision. In the first experiment, immediately prior to each testing session, participants either produced a strong calf muscle contraction in a fully plantarflexed (tiptoe) posture or underwent 15° dorsiflexion stretch. Contraction had no effect on task performance, whereas stretch produced a significant bias in ankle placement of 0.89 ± 0.6°, indicating that participants perceived their foot to be more plantarflexed compared to a control condition. In the second experiment, the right lower leg was cooled in iced water (≤ 5°C) for 10 minutes. Cooling increased joint matching error by ~0.4°, through a combination of increased bias and variability. These results confirm that conditioning the triceps surae muscles can alter perception of ankle joint position. Since body movement during quiet stance is in the order of 1°, the magnitude of these changes are relevant for balance.

## Introduction

During quiet standing, ankle joint motion accounts for a considerable proportion of postural sway [1]. Ankle proprioception is an important sensory input for balance, being more sensitive for perceiving sway than either visual or vestibular input [2]. Sources of ankle joint proprioception include pressure sensation from the foot sole, sensation within the joint itself, force transmitted through the Achilles tendon, and changes in calf muscle length. These are encoded by cutaneous afferents, joint capsule afferents, Golgi tendon organs and muscle spindles, respectively. Of these, muscle spindles are arguably the most important source of information for balance [3].

The ability of intrafusal muscle fibres to encode movement is affected by the surrounding extrafusal muscle. For example, when a voluntary contraction causes muscle shortening, intrafusal fibres slacken, impairing their ability to detect length changes. This is normally counteracted by simultaneous contraction of intrafusal fibres to maintain spindle sensitivity [4]. However, *passive* muscle changes may go undetected by the nervous system, causing uncompensated changes in spindle output. For example, the immediate history of muscle contraction influences its resting length, tension and stiffness [5,6], probably due to spontaneous formation of stable cross-bridges in passive muscle, increasing its stiffness when left still [7]. If the muscle is stretched, cross-bridges are broken and stiffness is reduced. These changes have proven consequences for joint angle perception in the upper limb; prior stretch of elbow flexors or extensors biases perceived arm position [8,9]. For example, following stretch of the right biceps muscle, the right elbow is perceived to be less flexed than the left elbow. Prior contraction has the opposite effect. If such history-dependent effects apply to the ankle joint, this could affect sensation of body orientation during quiet stance, with potential consequences for balance.

Temperature also affects spindle function. Cooling decreases spindle firing rates, with potential consequences for reflex function [10,11]. Studies of limb cooling on human proprioception, however, offer mixed results (see review by Costello & Donnelly [12]). LaRiviere & Osternig, (1994) reported no effect of lower leg cooling on ankle joint proprioception, although full proprioceptive acuity may have been limited by the flexed knee posture used in this study [13]. Another study did report a small deleterious effect of cooling during an ipsilateral ankle joint position reproduction task [14]. However, in this study only the foot itself was cooled. This raises the possibility that cooling might have a greater affect upon ankle proprioception if the musculature is cooled, and if sensation is tested under more ethological conditions i.e. a straight-legged, standing and weight-bearing posture.

A variety of techniques exist for assessing joint proprioception, each with their own advantages and disadvantages [15]. Here we use a joint position reproduction (JPR) task to determine the effects of prior stretch, contraction and cooling of the calf muscles upon proprioceptive acuity of the ankle joint. The JPR method has previously been used to demonstrate effects of muscle conditioning in the upper limb [5]. To assess proprioception in the lower limb, we employ a passive contralateral JPR method. We assess proprioception while upright and leaning against a stationary backboard. This ensures that the legs are straight and weight-bearing, to provide a mechanically optimal posture for ankle sensation. However, the use of the backboard removes the need to balance, ensuring that proprioceptive measures are not confounded by postural sway. Furthermore, the task is entirely passive from the perspective of the lower leg, ruling out active contributions to proprioception such as the use of motor command to estimate ankle position [16], or spindle modulation by alpha-gamma coactivation [4]. Hence, our experiment was deliberately engineered to test the influence of muscle conditioning on passive proprioceptive sensation, based solely upon peripheral feedback.

## Methods

### Participants

Ten volunteers (5 females) aged 22–43 participated in experiment 1. Twenty volunteers (10 females) aged 19–38 participated in experiment 2. Participants were recruited by email from the staff and student populate of the University of Birmingham between January and August of 2019. All participants were healthy and capable of unaided standing without pain or discomfort. Exclusion criteria included any neurological illness or leg injury which could affect standing or lower limb sensation. The research took place within the motor control laboratories of The School of Sport, Exercise & Rehabilitation Sciences, University of Birmingham. Experiments were approved by the local ethics committee within The School of Sport, Exercise and Rehabilitation Sciences, and were conducted in accordance with the declaration of Helsinki. All volunteers gave written informed consent.

### Apparatus

#### Ankle proprioception

**Fig 1:**
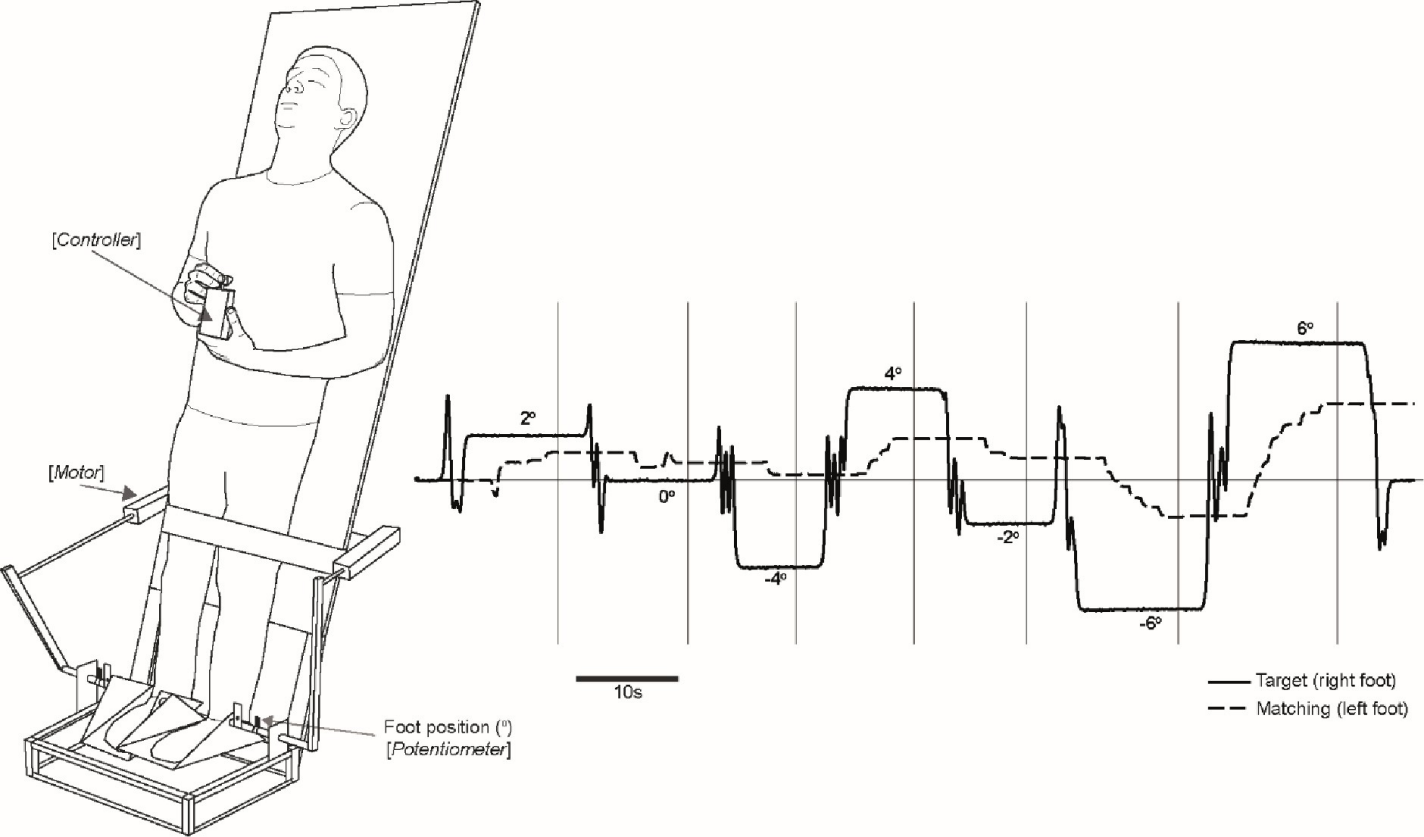
Ankle joint matching task. Participants leant against a board oriented 22° from vertical. Footplate positions were controlled by linear motors via levers. The target (right) foot was computer-controlled. The subject attempted to match its position by moving the matching (left) foot, using a hand-held dial controlled with their right hand. Representative position and torque traces are shown for a block of seven trials. Positive values refer to dorsiflexion position and plantarflexor torque. In each trial a small pseudo-random ‘wobble’ was introduced to the target foot immediately prior to settling into the new target position, in order to minimise memory-dependent effects. When the subject was content with their matching position they pressed a button. Button-press time points are depicted by vertical lines, which is when ankle position was measured. A strong tendency to underestimate the extent of ankle position can be seen in this subject.

Ankle proprioception was assessed using the apparatus depicted in Fig 1. Barefoot participants stood leaning backward against a board angled at 22° from vertical. Based upon the cosine of this lean angle, the proportion of body weight going through the legs was approximately 93%. This removed the need for balance, while ensuring the legs were weight-bearing. Participants were asked to stay relaxed and lean against the backboard, keeping their leg muscles as relaxed as possible. A strap around the lower thighs minimised movement of the knee joints, preventing the leg from buckling while they remained in position using minimal muscle activity. Each foot was placed upon a separate motorised footplate which could be independently rotated using two linear motors attached via levers (Model XTA3810S; Copley Motion Systems LLC, Basildon, UK). Footplate rotation axis was collinear with the ankle joint. Each footplate was instrumented with position sensors (Model CP-2UT; Midori Precisions Co., Tokyo, Japan).

#### Muscle cooling

In experiment 2 the right lower leg was cooled immediately prior to proprioceptive testing. A plastic container (300 x 375 x 490mm) was filled with iced water. While in a seated position, participants immersed their right leg into the container up to the knee. A neoprene wetsuit boot prevented foot pain and focussed the cooling primarily onto the muscles of the lower leg compartments. A water pump ensured continuous flow to avoid localised warming adjacent to the skin. Regular thermometer readings ensured water temperature remained ≤ 5°C. Ice was added if this temperature was exceeded.

#### Muscle twitch

Although skin temperature was monitored, we were unable to measure internal muscle temperature. To confirm that cooling affected the muscle we therefore measured muscle contractile properties in seven participants before and after cooling [17]. Participants sat barefoot with their lower leg in a vertical position and knee firmly clamped within a metal frame. An anode electrode (80x80 mm) was placed over the body of the gastrocnemius muscle, with a cathode placed ~100mm distally towards the Achilles tendon. Supramaximal direct current with a 50 μs pulse-width was applied to the triceps surae (DS7 current stimulator, Digitimer, UK). A strain gauge within the knee clamp registered the evoked plantarflexion force. Following determination of maximal evoked force, five twitches were recorded for analysis.

### Protocol

#### Proprioceptive testing

Participants performed a contralateral limb-matching task where they matched their left ankle position to their right ankle. The right ankle was moved to a pre-set target angle up to 6° in plantar- or +6° dorsiflexion, where an angle of 0° refers to the footplate being perpendicular to the back board. To minimise any memory-based performance effects due to the last trial position, the right footplate underwent a 2s period of random gentle dorsiflexion and plantarflexion motion prior to settling at its new position. This ‘wobble’ motion consisted of the sum of five sinewaves with frequencies randomly varying from 0.5–1.5Hz and an amplitude of <3°. A tone signalled the final position of the right foot and cued the participants to match the left ankle position using a hand-held knob to control the left footplate. When content with their matched position participants pressed a button that triggered the following trial. Progress was self-paced without time constraints. The eyes were closed at all times and no feedback of performance was provided.

#### Experiment 1 (stretch/contraction)

This experiment had three conditions performed within the same session. Immediately prior to each testing block, participants underwent a stretch or contraction of their right triceps surae, or no intervention (‘control’). Stretch was evoked by placing a 15° wedge underneath the participant’s right foot for 30s bringing the foot into dorsiflexion, while they were stood leaning against the backboard. 15° was chosen since this level of stretch could be comfortably sustained across a range of subjects. The experimenter then removed the wedge, and proprioceptive testing immediately ensued. For the contraction condition, participants stood with their right foot in maximum plantarflexion for 15s, with their left leg suspended. This was chosen as a maximum comfortable duration that all participants could sustain. Handrails were used to maintain balance but not to bear weight. They then lowered themselves into the apparatus when testing immediately ensued. Trials from each condition (stretch, contract, control) were grouped together, with the order randomised between participants.

Each testing block consisted of five target angles (-6°, -3°, 0°, 3°, 6°). Five blocks were included for each condition. In the first block, angles were presented in a random order. On subsequent blocks, the same sequence was used with the angle order shifted by one. This ensured all target angles were equally distributed throughout blocks. There were 25 trials per condition, making 75 trials in total.

#### Experiment 2 (cooling)

Joint matching ability was assessed immediately after right leg cooling (‘Cold’), and compared to performance under normal room-temperature conditions (‘Warm’). Cold and Warm sessions were performed on separate occasions 48 hours apart, with test order counter-balanced between participants. The warm session consisted of two practice blocks and six testing blocks. Within each block, one trial was performed at each of seven target angles (-6°, -4°, -2°, 0°, 2°, 4°, 6°) for a total of 42 test trials. Two-minute rest periods were given after the practice, and after blocks 2 and 4. The cold session was identical, except that the right leg was cooled for 10 minutes prior to the main test blocks and during each rest period. Surface skin temperature was measured from the anteromedial portion of the right ankle using tympanic thermometer immediately before the main test session, and at the start and end of each block.

### Data analysis

Joint matching performance was assessed via absolute error (AE), constant error (CE) and variable error (VE) (see chapter 2 in Schmidt & Lee [18]; Reynolds & Day [19]; Spray [28]). AE is the absolute difference between left and right target positions for each trial, reflecting overall performance. CE is the signed difference between left and right foot positions, reflecting bias. Positive values of CE mean that the responding (left) foot is more dorsiflexed compared to the target (right) foot. VE is the standard deviation of left foot placement for each target position, reflecting precision.

In the seven participants who underwent muscle stimulation, muscle relaxation time was measured as the time between peak and 50% twitch force.

In both experiments, separate repeated-measures ANOVAs were performed on each error measurement to determine effects of condition and target position. For experiment 1, this consisted of a two-factor ANOVA with three levels of condition (control, contract, stretch) and five levels of target (-6, -3, 0, 3, 6 degrees). For experiment 2, this consisted of a two-factor ANOVA with two levels of condition (warm, cold) and seven levels of target (-6, -4, -2, 0, 2, 4, 6 degrees).

The effect of the cooling protocol on skin temperature was assessed using a 2 Temperature (Warm, Cold) x 2 Period (Start-block, End-Block) repeated measures ANOVA. Significant main effects or interactions were further assessed with post-hoc comparisons with Sidak corrections. For participants undergoing muscle twitch testing, the effect of time point upon muscle relaxation time was analysed with one-way repeated-measures ANOVA. Statistical significance was set to p<0.05. All results in the main text are reported as mean ± standard deviation.

## Results

### Experiment 1—Stretch/Contraction

#### Joint matching error

**Fig 2:**
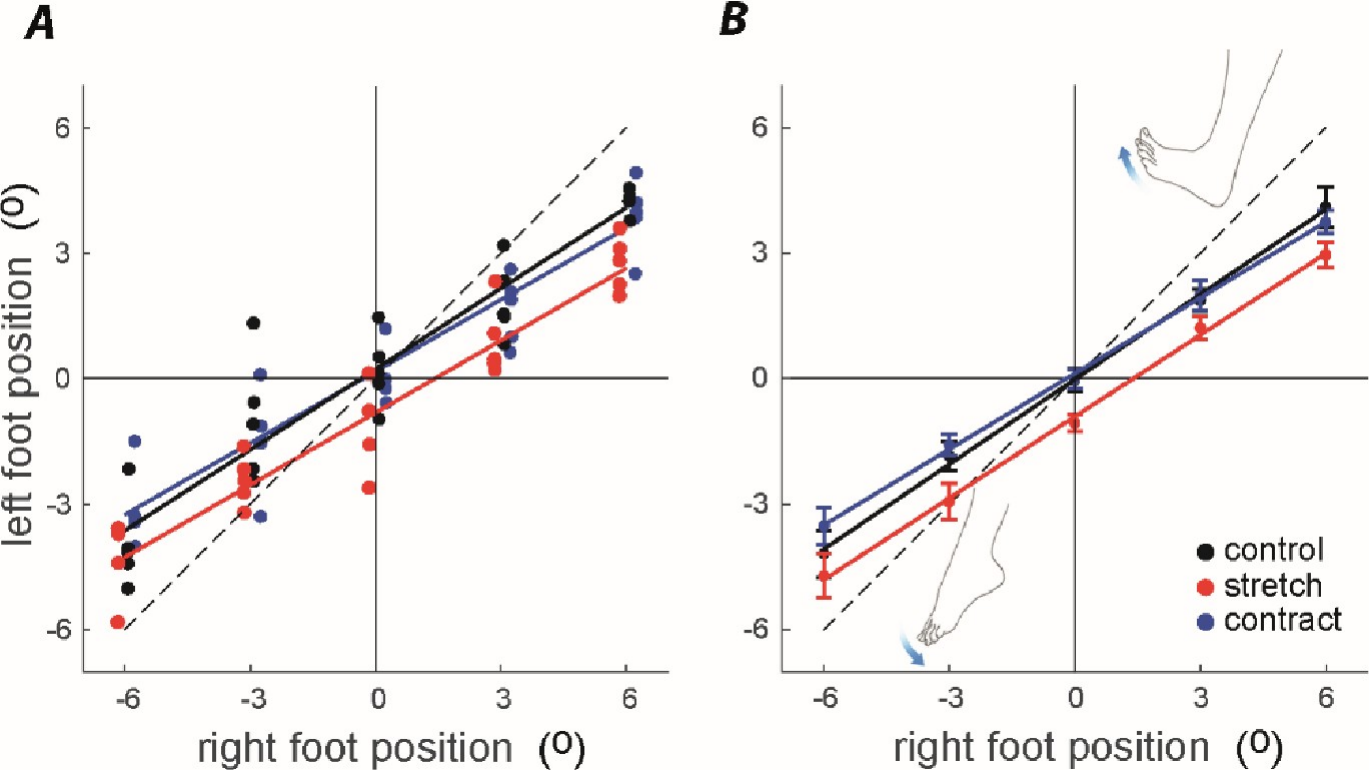
Effect of prior stretch & contraction upon mean foot placement. A) Representative data from a single participant. Data have been slightly offset along the x axis for improved visibility. The line of unity is shown by the dotted line, reflecting perfect performance. B) Mean data from 10 participants. Bars show one s.e.m.

Fig 2A shows representative foot matching performance from a single participant. For all conditions, this participant showed a strong tendency to underestimate ankle angle, such that their left ankle undershoots the right (target) ankle, particularly for larger angles. This is also apparent in the slopes of regression lines fitted to mean data in Fig 2B (normal = 0.67; contract = 0.60; stretch = 0.65). Left ankle position is clearly more planter-flexed during the stretch condition, for all target angles (mean difference from control = 0.89 ± 0.6°).

**Fig 3:**
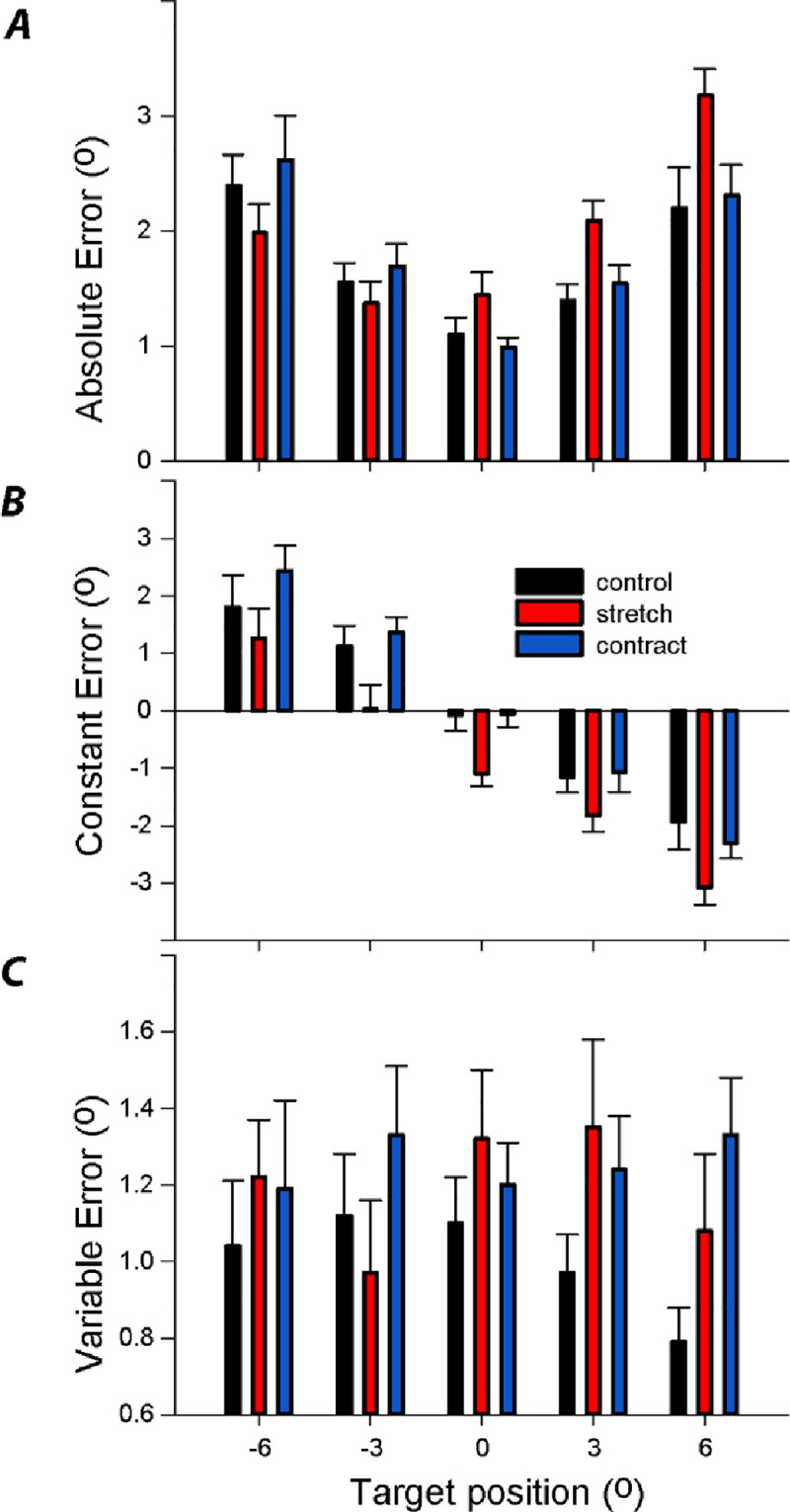
Effects of prior stretch and contraction upon ankle joint matching error. Bars show one s.e.m.

Absolute (AE), constant (CE) and variable error (VE) are shown in Fig 3. There was a significant interaction between condition and target for AE (Fig 3A; F_8,72_ = 6.5; p<0.001). This is due to stretch increasing AE in dorsiflexion and reducing AE in plantarflexion (see red bars raised at 6° and reduced at -6° target; Fig 3A).

The tendency to undershoot the target is reflected by positive CE values at the -6° target, reversing to negative values at +6° (Fig 3B). There was a significant effect of target position on CE (F_4,36_ = 35.6; p<0.001). There was also a significant effect of condition on CE (Fig 3B; F_2,18_ = 17.6; p<0.001). This is due to stretch causing a negative bias in CE compared to control or contraction (see red bars in Fig 3B; post-hoc comparisons; p ≤ 0.04). There was no interaction between condition and target (F_8,72_ = 1.2; p = 0.3). To determine if the effect of stretch upon CE was affected by trial order, we averaged errors separately for the first and last trials in each block, averaging across targets. There was no significant main effect of order upon CE (F_1,9_ = 3.1; p = 0.11). Furthermore, there was no interaction between order and condition (F_1,9_ = 0.74; p = 0.50).

For VE, there was no main effect of condition (F_2,18_ = 1.7; p = 0.21) or target (F_4,36_ = 0.44; p = 0.78), nor an interaction of condition and target (Fig 3C; F_8,72_ = 1.2; p = 0.27).

Overall, the results indicate that stretch caused a significant bias in foot placement, without affecting precision.

### Experiment 2—Cooling

#### Skin temperature and muscle contractile properties

**Fig 4:**
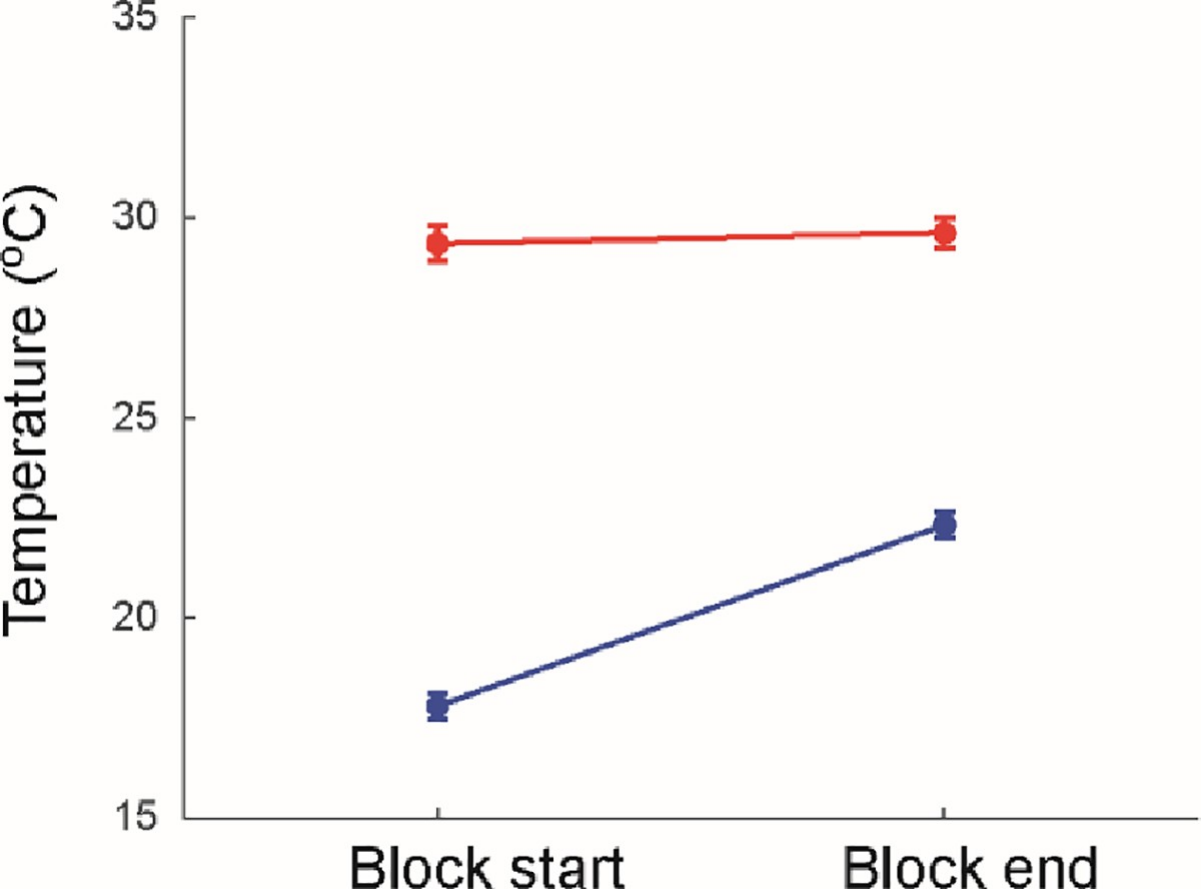
Effect of cooling upon ankle skin temperature. The cold condition (blue) involved immersion of the right lower leg into iced water, while the warm condition (red) involved rest at room temperature. Data show mean values for the beginning and end of each proprioceptive testing block; (see temperature measurement points in Fig 5). Bars show one s.e.m.

A comparison of skin temperatures at the start and end of testing blocks revealed an interaction of condition and time point (Fig 4; F_1,19_ = 320; p<0.001). At the start of the test blocks, cooling reduced skin temperature to 17.8 ± 1.4°C as compared to 29.4 ± 2.0°C for the warm condition (p<0.001). Although skin temperature increased slightly to 22.3 ± 1.4°C during the cold test blocks, this remained significantly lower than the 29.6 ± 1.7°C recorded at the end of the warm test blocks (p<0.001).

**Fig 5:**
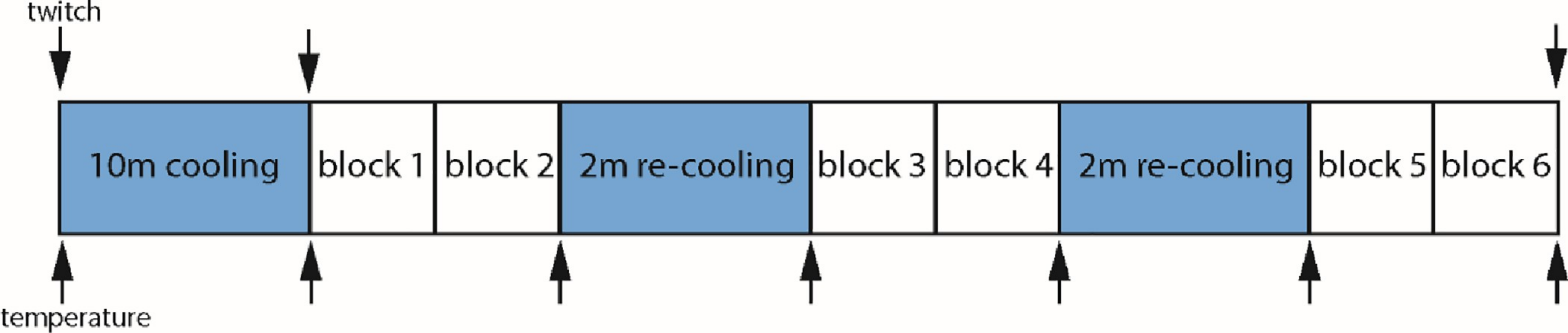
Cooling protocol. The right lower leg was cooled in iced water for 10 minutes immediately prior to the first testing block. Each block included 7 trials. Re-cooling periods were included to keep the leg cold. Upward-pointing arrows indicate times when skin temperature was recorded in all participants. In seven participants, muscle twitch properties were also investigated at time points represented by downward-pointing arrows. Each twitch session lasted 10 minutes. The protocol for the warm session was the same, but with rest periods instead of cooling. Both warm and cold sessions were preceded by two practice blocks, not shown above.

Seven participants underwent muscle twitch measurements at the three time points shown in Fig 5. Twitch relaxation time significantly increased from 82 ± 10 ms (warm) to 110 ± 16ms (cold 1) to 127 ± 22ms (cold 2) (F_2,12_ = 41; p<0.001), confirming that cooling affected muscle contractile properties.

#### Joint matching error

**Fig 6:**
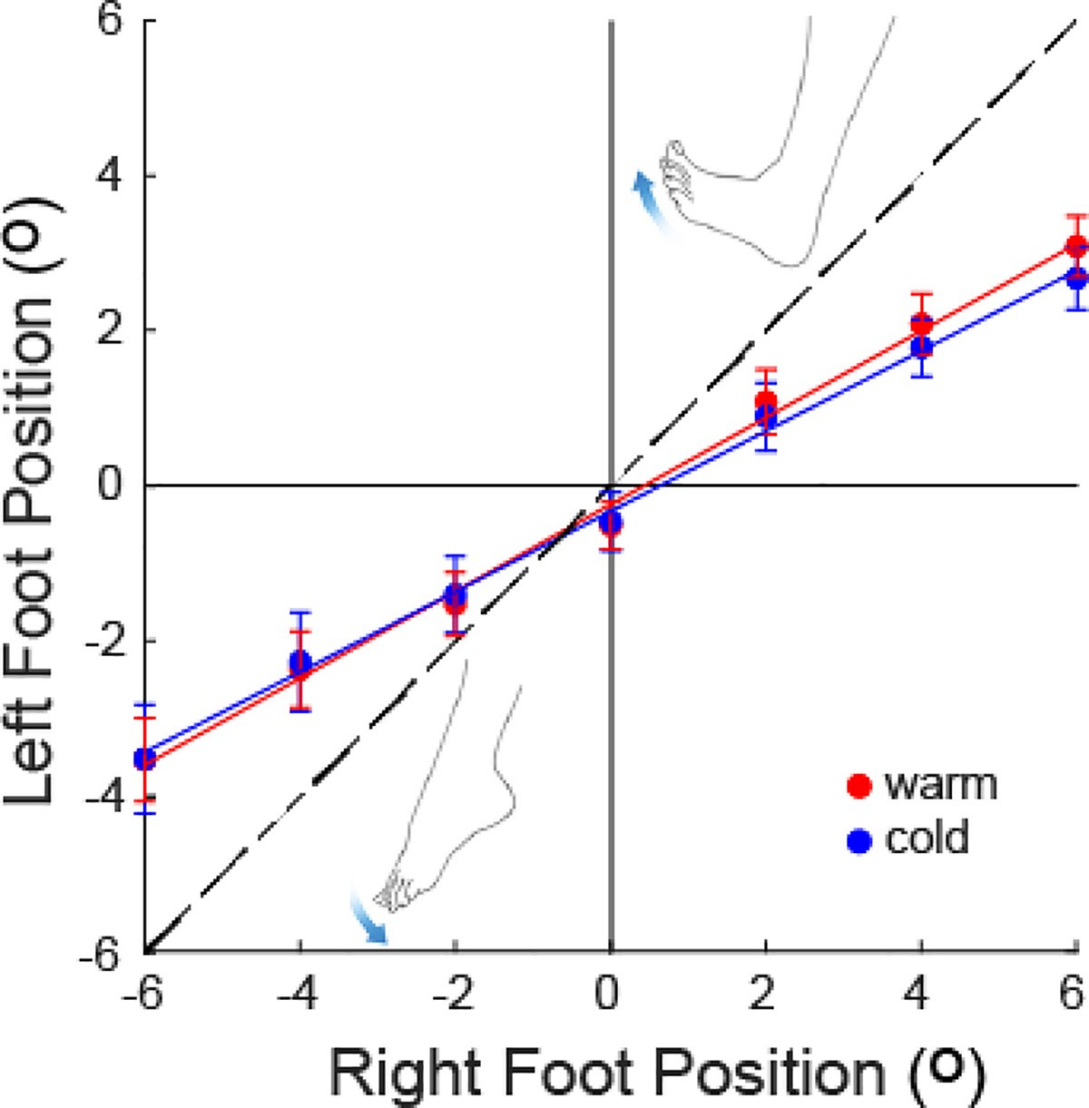
Effects of cooling upon mean foot placement. Bars show one s.e.m.

The mean foot placement across all participants is shown in Fig 6. As before, for larger target angles there was a strong tendency to undershoot the target. There is also a slight tendency for cooling to produce a plantarflexion bias.

**Fig 7:**
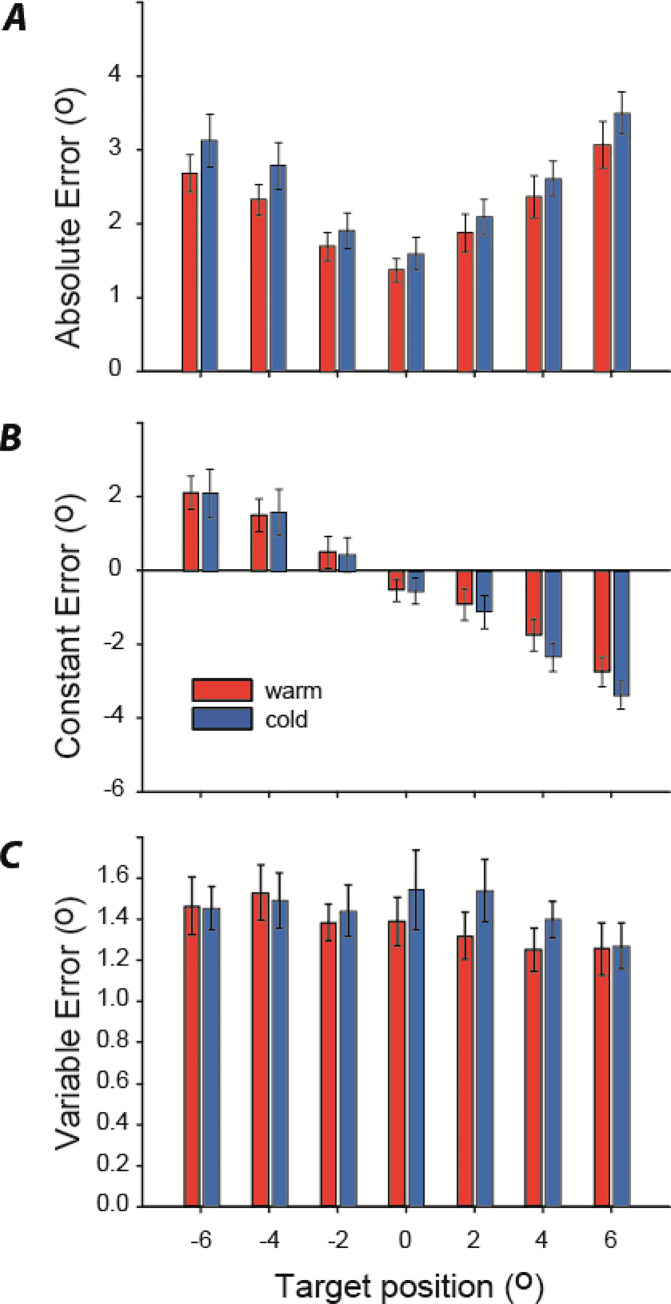
Effects of cooling upon foot placement error. Bars show one s.e.m.

Absolute, constant and variable error are shown in Fig 7. AE was significantly greater for the cold condition, by an average of 0.32° (Fig 7A; main effect of temperature: F_1,19_ = 4.6; p = 0.045). Furthermore, there was a significant main effect of target position upon AE, reflecting larger error at more extreme angles (Fig 7A; F_6,114_ = 9.7; p<0.001). To determine if the effect of cooling upon AE was attenuated over time, we compared mean errors on the first and second testing blocks following iced water treatment (i.e. blocks 1, 3, 5 vs blocks 2, 4, 6 in Fig 5). There was no main effect of order (F_1,19_ = 0.29; p = 0.6), nor any interaction between order and condition (F_1,19_ = 2.25; p = 0.15).

There was no effect of cooling upon CE (Fig 7B; F_1,6_ = 1.2; p = 0.66) or VE (Fig 7C; F_1,19_ = 1.1; p = 0.32). However, there was a significant effect of target position upon CE (Fig 7B; F_6,114_ = 80.3; p<0.001), again reflecting larger errors at more extreme angles.

## Discussion

We measured proprioceptive acuity of the ankle joint using a contralateral joint matching task. The ability to voluntarily match the left ankle angle to the perturbed right ankle angle was significantly affected by interventions which altered the mechanics and/or physiology of the triceps surae muscles. Prior stretch caused a perceptual bias, causing participants to perceive their ankle to be more plantarflexed than normal. Cooling, however, did not produce a significant perceptual bias but did increase overall joint matching error. These effects, likely mediated by changes in muscle spindle output, may have consequences for balance during quiet stance, a task for which ankle proprioception is probably the most important sensory input [3]. The magnitude of the effects we observed may seem small (0.4–1° error). However, they are relevant for balance, since RMS ankle joint motion is around 0.25–0.45° during quiet standing, with a range of < 2° [1]. Hence, a proprioceptive uncertainty of 0.4-1° could affect postural control during quiet stance, and possibly during the stance phase of gait also.

Participants were capable of differentiating between the various ankle positions for both experiments, as confirmed by the significant effect of target position upon foot placement. However, they showed a general tendency to underestimate the extent of joint movement, irrespective of condition. This was most pronounced for larger angles (± 6°), where the left foot was placed approximately half as far as the right foot (e.g. Fig 6). The joint matching task we employed involves sensation from both limbs; first the participant must judge the position of the target (right) leg and second, they must then judge the position of the responding (left) leg. Any tendency to underestimate ankle rotation would likely be similar for both legs. Hence, the considerable misjudgement we observed is likely a sum of the perceptual errors from both legs [20]. Absolute and constant errors were lowest when attempting to match an ankle angle of 0°, where the footplate is perpendicular to the backboard. This suggests that proprioceptive acuity may be tuned to those angles most commonly experienced during quiet stance, as previously suggested for the upper limb [21,22].

The effect of stretch can be attributed to changes in the length of the triceps surae muscle which, in turn, alters spindle output. Thixotropic properties of muscle tissue mean that its stiffness, resting tension and slack length depend on its immediate history of movement [7,23]. Stretching reduces muscle stiffness and resting tension, and increases slack length when the limb is returned to its original position. These factors affect intrafusal as well as extrafusal muscle fibres, causing a reduction in spindle firing rate [5,24]. This can explain the altered perception of joint position reported here. Fifteen degrees of stretch would be expected to reduce calf muscle spindle firing rate when the ankle is subsequently positioned at the test angles. Hence, this signal will be attenuated during ankle rotation, causing the perception that the muscle is shorter than it actually is (see Fig 2). Previous studies have demonstrated increased sway following calf muscle stretch [25]. Hence, the altered ankle proprioception we observed may have consequences for balance. This deleterious effect of muscle stretching does not happen following alternated stretching of both calf muscles and tibialis anterior [26]. This is consistent with our findings, since any proprioceptive bias which might lead to increased sway could be negated by simultaneous influences on both agonist and antagonist muscle spindle output.

Prior contraction should theoretically have the opposite effect, as previously shown for the upper limb [27]. However, strong voluntary contraction of the calf muscle had no influence on ankle proprioceptive acuity. This discrepancy between stretch and contraction could be due to inherent differences between the upper and lower limbs, or methodological issues. For example, here participants were stood upright in a passive but partially weight-bearing posture. Adopting this posture immediately after the voluntary calf muscle contraction may have been sufficient to stretch the muscle beyond its thixotropic limit. We deliberately set out to maximise calf muscle shortening, and therefore any proprioceptive effects, by asking participants to adopt a tiptoe posture during the plantarflexion contraction. However, there was likely to be some cocontraction of the antagonist Tibialis Anterior (TA) during the plantarflexing contraction. This contraction may have negated any effects induced by calf muscle contraction. Alternatively, the ‘wobble’ motion preceding all trials may have been enough to loosen the muscle, despite being small in amplitude. However, if this were the case one might expect stretch also to have had no effect, since the muscle would be in a permanent state of low stiffness. In either case, these results demonstrate asymmetrical effects of contraction and stretch upon ankle proprioception during standing.

Cooling had a more subtle effect than stretch. Nevertheless, it caused a statistically significant increase in absolute error of ~0.4°, reflecting poorer ankle matching performance. This could not be directly attributed either to a decrease in precision (VE), or a change in bias (CE) alone, but must reflect a combination of the two since AE is a combination of CE and VE [28]. How could cooling produce such changes? Animal studies demonstrate a linear increase in spindle firing rate with increasing temperature [10,11]. For the ankle joint matching task, this would reduce the extent to which spindles fire with increasing plantarflexion when cooled, and this might be expected to cause a directional bias in the perception of joint angle. However, simultaneous cooling of the antagonist Tibialis Anterior muscle would have also occurred when immersing the lower limb in iced water. The TA has been postulated to play a role in ankle proprioception equally important to that of the triceps surae [29]. Hence, if agonist-antagonist muscle pairs exhibited similar changes in the spindle rate coding of joint angle, these effects might cancel out. This could explain why we observed no consistent perceptual bias caused by cooling.

Calf muscle spindles arguably provide the main contribution to ankle proprioception during standing [3]. However, in our experiment, toes-up rotation of the footplate would inevitably encounter some passive resistance, in addition to positional change, due to the elasticity of the triceps surae and Achilles tendon. This resistance would be detected by pressure and/or force sensors, including golgi tendon organs (GTO’s) and foot sole pressure afferents. The effects of cooling and stretch might be explained by these changes in the function of these receptors. Since participants wore a wetsuit boot during the ice immersion it is unlikely that the foot was significantly cooled. Therefore pressure sensation on the sole was probably unaffected. In contrast, the musculo-tendinous junction of the triceps surae, where GTO’s are clustered, *was* exposed to iced water. However, the effect of temperature upon GTO’s is much less pronounced than its effect upon spindles, especially during cooling [11]. Nevertheless, their potential role in mediating temperature-dependent effects upon proprioception cannot be excluded. Stretching muscle certainly produces changes in spindle firing rates when returned to its original length [9]. After-effects of muscle stretch upon joint position sense have been largely attributed to this change [5]. Again, however, the role of GTO’s cannot be excluded.

Our proprioception task has inherent limitations. Participants stood passively while leaning against a backboard. We deliberately engineered the task in this way to assess proprioception unaffected by motor output. While this may better reflect the performance of peripheral sensors (e.g. muscle spindles), it is obviously different to normal standing. Quiet stance is characterised by constant active adjustments in calf muscle length [30]. This motor output, combined with the increased stiffness of a contracting muscle, will alter the way muscle spindles encode joint angle. Inevitably, this makes the relationship between joint angle and sensory input complicated [29,31]. However, by reducing the task to a static, passive positional judgement, it seems likely that our observed effects can be attributed to changes in peripheral sensory input alone. We asked participants to rotate their left leg until it felt matched to the right. Although we did not assess handedness, it is likely that the majority were right-handed. There is some evidence to suggest better proprioceptive acuity in the non-dominant side of the body, across multiple joints (i.e. better proprioception on the left side of the body for right-handed participants) [32]. Hence, it is possible that better judgements were observed than if we had used the right leg. However, as stated above, our task requires sensory input from both sides of the body, so may not be affected by laterality in the same way.

Another limitation may be the generalisability of our technique. Here we used a passive contralateral JPR task to assess relative position sensation between the left and right limbs. Variations on the JPR method include active movement of the limbs, and ipsilateral matching, whereby the same limb is used to return to a memorised position. Methods other than JPR include measuring the threshold to detection of passive motion (TTDPM), and active movement extent discrimination (AMEDA) [15]. TTDPM assesses movement, rather than position sensation, which may be pertinent for postural control [33]. Like JPR, AMEDA assesses position sensation but, unlike our JPR test, it involves active positioning of the limb. This may better reflect natural standing conditions. Perhaps surprisingly, it has been shown that there is virtually no correlation between these tests [34,35]. This suggests that proprioceptive acuity is multifaceted and cannot be reduced to a single performance variable [36]. Nevertheless, the advantages of our method include its minimal cognitive requirements, and the pure assessment of peripheral sensation, uncontaminated by motor output. Most importantly, it is ideally suited for assessing the effects of muscle conditioning interventions, with the unaffected limb acting as a control for comparison against the affected limb.

In summary, we have shown that ankle joint position sense can be influenced by conditioning the triceps surae muscle. Prior muscle stretch produced a systematic bias in the perception of ankle angle, whereas cooling produced an overall increase in positioning error. It is likely that both effects can be attributed to changes in spindle function. Future research will determine if these changes in proprioceptive function have implications for postural control and fall risk.

## References

[1] Gatev P, Thomas S, Kepple T, Hallett M. Feedforward ankle strategy of balance during quiet stance in adults. J Physiol. 1999;514 (Pt 3: 915–928. 9882761 10.1111/j.1469-7793.1999.915ad.x PMC2269093

[2] Fitzpatrick R, McCloskey DI. Proprioceptive, visual and vestibular thresholds for the perception of sway during standing in humans. J Physiol. 1994;478 (Pt 1: 173–186. 7965833 10.1113/jphysiol.1994.sp020240 PMC1155655

[3] Fitzpatrick R, Rogers DK, McCloskey DI. Stable human standing with lower-limb muscle afferents providing the only sensory input. J Physiol. 1994;480 (Pt 2: 395–403. 7869254 10.1113/jphysiol.1994.sp020369 PMC1155855

[4] Hunt CC, Kuffler SW. Stretch receptor discharges during muscle contraction. J Physiol. 1951;113: 298–315. 10.1113/jphysiol.1951.sp004573 14832776 PMC1392996

[5] Proske U, Gandevia SC. The proprioceptive senses: their roles in signaling body shape, body position and movement, and muscle force. Physiol Rev. 2012;92: 1651–1697. 10.1152/physrev.00048.2011 23073629

[6] Sakanaka TE, Lakie M, Reynolds RF. Sway-dependent changes in standing ankle stiffness caused by muscle thixotropy. J Physiol. 2015 [cited 23 Dec 2015]. 10.1113/JP271137 26607292 PMC4988472

[7] Campbell KS, Lakie M. A cross-bridge mechanism can explain the thixotropic short-range elastic component of relaxed frog skeletal muscle. J Physiol. 1998;510 (Pt 3: 941–962. 10.1111/j.1469-7793.1998.941bj.x PMC2231083 9660904

[8] Proske U, Morgan DL, Gregory JE. Thixotropy in skeletal muscle and in muscle spindles: a review. ProgNeurobiol. 1993;41: 705–721. 10.1016/0301-0082(93)90032-n 8140258

[9] Gregory JE, Morgan DL, Proske U. Aftereffects in the responses of cat muscle spindles and errors of limb position sense in man. J Neurophysiol. 1988;59: 1220–1230. 10.1152/jn.1988.59.4.1220 3373276

[10] Eldred E, Lindsley DF, Buchwald JS. The effect of cooling on mammalian muscle spindles. Exp Neurol. 1960;2: 144–157. 10.1016/0014-4886(60)90004-2 13819871

[11] Mense S. Effects of temperature on the discharges of muscle spindles and tendon organs. Pflügers Arch Eur J Physiol. 1978;374: 159–166. 10.1007/BF00581297 566425

[12] Costello JT, Donnelly AE. Cryotherapy and joint position sense in healthy participants: A systematic review. Journal of Athletic Training. 2010 pp. 306–316. 10.4085/1062-6050-45.3.306 20446845 PMC2865970

[13] Refshauge KM, Fitzpatrick RC. Perception of movement at the human ankle: effects of leg position. J Physiol. 1995;488: 243–248. 10.1113/jphysiol.1995.sp020962 8568660 PMC1156717

[14] Hopper D, Whittington D, Davies J. Does ice immersion influence ankle joint position sense? Physiother Res Int. 1997;2: 223–236. 10.1002/pri.108 9408933

[15] Han J, Waddington G, Adams R, Anson J, Liu Y. Assessing proprioception: A critical review of methods. J Sport Heal Sci. 2016;5: 80–90. 10.1016/J.JSHS.2014.10.004 30356896 PMC6191985

[16] Gandevia SC, Smith JL, Crawford M, Proske U, Taylor JL. Motor commands contribute to human position sense. J Physiol. 2006;571: 703–710. 10.1113/jphysiol.2005.103093 16439427 PMC1805798

[17] Davies CTM, Mecrow IK, White MJ. Contractile properties of the human triceps surae with some observations on the effects of temperature and exercise. Eur J Appl Physiol Occup Physiol. 1982;49: 255–269. 10.1007/BF02334074 6889502

[18] Schmidt RA, Lee TD. Motor control and learning: A behavioral emphasis. Human Kinetics_ _; 1999.

[19] Reynolds RF, Day BL. Visual guidance of the human foot during a step. J Physiol. 2005;569: 677–684. 10.1113/jphysiol.2005.095869 16179363 PMC1464243

[20] Goble DJ. Proprioceptive Acuity Assessment Via Joint Position Matching: From Basic Science to General Practice. Phys Ther. 2010;90: 1176–1184. 10.2522/ptj.20090399 20522675

[21] Gritsenko V, Krouchev NI, Kalaska JF. Afferent Input, Efference Copy, Signal Noise, and Biases in Perception of Joint Angle During Active Versus Passive Elbow Movements. J Neurophysiol. 2007;98: 1140–1154. 10.1152/jn.00162.2007 17615137

[22] Fuentes CT, Bastian AJ. Where is your arm? Variations in proprioception across space and tasks. J Neurophysiol. 2010;103: 164–171. 10.1152/jn.00494.2009 19864441 PMC4116392

[23] Stubbs PW, Walsh LD, D’Souza A, Héroux ME, Bolsterlee B, Gandevia SC, et al History-dependence of muscle slack length following contraction and stretch in the human vastus lateralis. J Physiol. 2018;596: 2121–2129. 10.1113/JP275527 29604053 PMC5983182

[24] Wood SA, Gregory JE, Proske U. The influence of muscle spindle discharge on the human H reflex and the monosynaptic reflex in the cat. J Physiol. 1996;497: 279–290. 10.1113/jphysiol.1996.sp021767 8951729 PMC1160930

[25] Nagano A, Yoshioka S, Hay DC, Himeno R, Fukashiro S. Influence of vision and static stretch of the calf muscles on postural sway during quiet standing. Hum Mov Sci. 2006;25: 422–434. 10.1016/j.humov.2005.12.005 16563540

[26] Yeomans MA, Nelson AG, MacLellan MJ, Hondzinski JM. Visually-guided saccades attenuate postural sway under non-fatigued, fatigued, and stretched states. Exp Brain Res. 2018;236: 3351–3361. 10.1007/s00221-018-5384-2 30259110

[27] Winter JA, Allen TJ, Proske U. Muscle spindle signals combine with the sense of effort to indicate limb position. J Physiol. 2005;568: 1035–1046. 10.1113/jphysiol.2005.092619 16109730 PMC1464181

[28] Spray JA. Absolute error revisited: An accuracy indicator in disguise. J Mot Behav. 1986;18: 225–238. 10.1080/00222895.1986.10735379 15136281

[29] Di Giulio I, Maganaris CN, Baltzopoulos V, Loram ID. The proprioceptive and agonist roles of gastrocnemius, soleus and tibialis anterior muscles in maintaining human upright posture. J Physiol. 2009;587: 2399–2416. 10.1113/jphysiol.2009.168690 19289550 PMC2697307

[30] Loram ID, Maganaris CN, Lakie M. Active, non-spring-like muscle movements in human postural sway: how might paradoxical changes in muscle length be produced? J Physiol. 2005;564: 281–293. 10.1113/jphysiol.2004.073437 15661825 PMC1456051

[31] Loram ID, Maganaris CN, Lakie M. Paradoxical muscle movement in human standing. J Physiol. 2004;556: 683–689. 10.1113/jphysiol.2004.062398 15047776 PMC1664994

[32] Han J, Anson J, Waddington G, Adams R. Proprioceptive performance of bilateral upper and lower limb joints: Side-general and site-specific effects. Exp Brain Res. 2013;226: 313–323. 10.1007/s00221-013-3437-0 23423167 PMC3627017

[33] Pai YC, Patton J. Center of mass velocity-position predictions for balance control. JBiomech. 1997;30: 347–354. 9075002 10.1016/s0021-9290(96)00165-0

[34] Yang N, Adams RD, Han J, Waddington G. Fechner’s three experimental methods: Do the results correlate? In: Muller F, Ludwigs L, Kupper M, editors. Proceedings of the 34th Annual Meeting of the International Society for Psychophysics. 2018. pp. 161–166. Available: http://proceedings.fechnerday.com/index.php/proceedings/article/view/616/603#page=175

[35] Yang N, Waddington G, Adams R, Han J. Joint position reproduction and joint position discrimination at the ankle are not related. Somatosens Mot Res. 2020; 1–9. 10.1080/08990220.2020.1746638 32281906

[36] Krewer C, Van de Winckel A, Elangovan N, Aman JE, Konczak J. Commentary on: “Assessing proprioception: A critical review of methods” by Han et al. Journal of Sport and Health Science. Elsevier B.V.; 2016 pp. 91–92. 10.1016/j.jshs.2015.11.001 30356516 PMC6188602

